# Single-cell analysis reveals corticosteroid-associated impairment of tumor-infiltrating NK cells in glioblastoma patients

**DOI:** 10.1038/s41598-026-52528-1

**Published:** 2026-05-21

**Authors:** Simone Balin, Paolo Marzano, Alessandro Limonta, Sara Terzoli, Matteo Simonelli, Lorenzo Bello, Federico Pessina, Benedetta Savino, Joanna Mikulak, Francesca Calcaterra, Elena Fontana, Massimo Locati, Domenico Mavilio, Silvia Della Bella

**Affiliations:** 1 Unit of Clinical and Experimental Immunology, IRCCS Humanitas Research Hospital, Rozzano, Milan Italy; 2 Unit of Clinical and Experimental Immunology, Department of Medical Biotechnology and Translational Medicine, University of Milan, via Fratelli Cervi 93, Segrate, 20054 Milan Italy; 3 Fondazione Human Technopole, Milan, Italy; 4 Department of Biomedical Sciences, Humanitas University, Pieve Emanuele, Milan, Italy; 5 Department of Medical Oncology and Hematology, IRCCS Humanitas Research Hospital, Rozzano, Milan Italy; 6 Unit of Oncological Neurosurgery, IRCCS Istituto Ortopedico Galeazzi, Milan, Italy; 7 Department of Oncology and Hemato-Oncology, University of Milan, Milan, Italy; 8 Department of Neurosurgery, IRCCS Humanitas Research Hospital, Rozzano, Milan Italy; 9 Laboratory of Leukocyte Biology, Department of Medical Biotechnology and Translational Medicine, University of Milan, Segrate, Milan Italy; 10 Laboratory of Leukocyte Biology, IRCCS Humanitas Research Hospital, Rozzano, Milan Italy

**Keywords:** Glioblastoma, NK cells, Perioperative corticosteroids, Single cell-RNA sequencing, Cancer, Immunology, Neuroscience, Oncology

## Abstract

Glioblastoma is the most common and aggressive malignant primary brain tumor in adults, characterized by poor prognosis and limited response to current therapeutic strategies. Corticosteroids, particularly dexamethasone, are widely used in clinical practice to control symptomatic peritumoral edema, yet they exert profound immunosuppressive effects whose impact on tumor-infiltrating NK cells remains poorly defined. To address this issue, we analyzed a publicly available single-cell RNA sequencing dataset of CD45⁺ cells isolated from five IDH-wildtype glioblastomas, including two patients undergoing perioperative dexamethasone treatment and three untreated patients, alongside two non-tumor brain samples. Our results suggested that perioperative dexamethasone exposure was associated with a reshaped intratumoral NK cell landscape, characterized by a relative enrichment of CD56^bright^ subsets. Perioperative corticosteroid treatment was also associated with transcriptional features consistent with impaired NK cell effector programs, as evidenced by reduced cytotoxicity and inflammation scores, down-regulation of granzymes, perforin, pro-inflammatory cytokines, and activating receptors, and concomitant upregulation of inhibitory molecules. Gene set enrichment analysis further demonstrated strong downregulation of NK cell-mediated cytotoxicity and cell-killing pathways. Together with previous evidence of corticosteroid-associated impairment of glioma-infiltrating dendritic cells, these findings suggest that perioperative steroid therapy may affect both cytotoxic and antigen-presenting innate compartments. If confirmed in larger patient cohorts, these observations may support the importance of minimizing steroid exposure to optimize immunotherapeutic efficacy.

**Supplementary Information:**

The online version contains supplementary material available at 10.1038/s41598-026-52528-1.

## Introduction

Gliomas are the most common malignant primary brain tumors in adults, accounting for nearly 75% of cases, and remain among the most challenging cancers to treat^1^. The 2021 WHO classification, which integrates both histological and molecular genetic features, has reshaped how these tumors are defined. Within this framework, adult-type diffuse gliomas are categorized as isocitrate dehydrogenase (IDH)-mutant astrocytomas, IDH-mutant and 1p/19q-codeleted oligodendrogliomas, and IDH-wildtype (WT) glioblastoma, which represents the subgroup with the poorest clinical outcome^2^. Despite complete tumor resection followed by chemoradiation, most patients with IDH-wildtype glioblastoma do not survive beyond 12–15 months^3^.

The lack of effective therapeutic options has driven a strong interest in immunotherapeutic approaches. Nevertheless, strategies such as immune checkpoint blockade and peptide vaccination have so far failed to prolong survival in glioblastoma patients, mainly because of the low immunogenicity of these tumors and the highly suppressive tumor microenvironment (TME)^4^. In this scenario, a deep characterization of immune cell subsets within the glioblastoma TME has become essential to improve our comprehension of the mechanisms that hamper effective antitumor immunity in these patients.

Natural killer (NK) cells are key cytotoxic effectors of the innate immune system, endowed with the ability of eliminating malignant and virus-infected cells without prior antigen sensitization^5^. Their role in glioblastoma, however, remains incompletely defined. Whereas preclinical studies have shown that NK cells can efficiently kill glioma cells, evidence from human tumors indicates that NK cells are numerically scarce and functionally impaired in the glioblastoma TME. Several mechanisms, including altered expression of NK-activating ligands on glioma cells, increased levels of inhibitory molecules, and the presence of immunosuppressive cytokines, have been demonstrated to contribute to this NK cell dysfunction^6–8^.

A further layer of complexity is introduced by the widespread use of corticosteroids in glioblastoma patients. Steroids are routinely administered to control symptomatic peritumoral edema, though they are known to exert potent immunosuppressive effects by acting on multiple lymphoid and myeloid cell types^9^. Despite their extensive clinical use, the impact of corticosteroid therapy on glioma-infiltrating NK cells remains poorly defined.

In this study, we leveraged a public single-cell RNA sequencing dataset obtained on IDH-WT glioblastoma tissues from treatment-naïve patients and patients receiving perioperative corticosteroids. We investigated the abundance, transcriptional state and programs of tumor-infiltrating NK cells in relation to steroid exposure. This comparative analysis provides new insights into the plausible impact of a standard supportive therapy on glioblastoma-associated innate immune responses, with potential implications for the design and efficacy of therapeutic interventions.

## Methods

### scRNA-seq data processing

Single cell original data have been downloaded from the Zenodo Repository (RRID:SCR_004129) under the accession number of 10.5281/zenodo.6046299. The study protocol was approved by the Institutional Review Boards of Humanitas Research Hospital (ONC-OSS-04-2017; 29/19), and written informed consents were provided by all participants before inclusion in the study in compliance with the Declaration of Helsinki.

The gene expression count matrices of 2 wild-type DEX-treated, 3 wild-type untreated patients and from 2 healthy brain tissues were extracted from the original dataset, imported in Python (v3.9.7, RRID:SCR_008394), and analyzed with Scanpy (v1.9.3, RRID:SCR_018139)^10^. None of the patients received radiotherapy or chemotherapy before surgery. DEX-treated patients were receiving dexamethasone 6 mg/day and 4 mg/day, respectively, until the day of surgery. Raw data matrices of all samples (n = 7) were merged in one single AnnData object (v0.10.4, RRID:SCR_018209)^11^, and cells containing > 200 genes and ≤ 10% mitochondrial genes were kept for downstream analysis. Gene expression matrices were then log-normalized with a scale factor of 10000. Highly variable genes (HVGs, n = 4000) were identified across each sample (batch_key = ‘sample_ID’) using the Seurat_v3 dispersion-based methods on raw counts. Principal component analysis (PCA) was performed using HVGs via ARPACK implementation of singular value decomposition. To limit the risk of biases, multiple sample-data integration was performed using the harmonypy package (v0.0.10, RRID:SCR_022798)^12^, setting ‘sample_ID’ as key covariate in the formula. Neighbors (n = 15) were identified using the top 30 components of the Harmony-corrected PCA space, and clustering was performed using the Leiden algorithm^13^, and clusters were then embedded in two dimensions UMAP^14^, using the Harmony-corrected PCA space, as previously described^15^. For the re-clustering of cells of interest, we extracted cells from the total dataset, re-computed the HVGs on raw counts, and re-integrated the samples with the same settings reported above.

### Differential abundance analysis

To assess whether DEX treatment was associated with changes in NK cell subset composition, differential abundance analysis of NK cells was performed using the Milopy (v0.1.1, RRID:SCR_025630)^16^ algorhitm. A k-nearest neighbor (kNN) graph was first computed on the Harmony-corrected PCA embedding, using 21 neighbors per cell, to ensure that each neighborhood contained approximately 3 cells from each sample. Cell neighborhoods were then defined with Milopy using a 10% sampling proportion to generate representative and non-redundant local neighborhoods across the transcriptional manifold. Each neighborhood was then annotated according to its predominant NK cell cluster identity, based on the clustering annotation. For each neighborhood, cell counts per sample were aggregated and tested for differential abundance between DEX-treated and untreated conditions using a generalized linear model with the design ~Treatment and the contrast DEX-treated vs untreated. The resulting log fold changes (logFCs) and FDR-adjusted p-values were used to identify neighborhoods significantly enriched or depleted upon treatment. Results were represented in boxplots that summarize the distribution of logFC values across Milopy neighborhoods assigned to each cluster.

### Differentially expressed genes analysis

Differentially expressed genes (DEGs) were identified using the Wilcoxon rank-sum test with the Benjamini-Hochberg correction method implemented in the *scanpy.tl.rank_genes_groups()* function to identify cluster specific genes and to compare CD56^bright^ and CD56^dim^ NK cells between DEX-treated and untreated patients. Genes were considered significantly differentially expressed if they met the criteria of adjusted p-value < 0.05 and |Log_2_-FoldChange| > 0.25. Results were visualized as volcano plots using the EnhancedVolcano Bioconductor package (v1.22.0, RRID:SCR_018931).

### Functional enrichment analysis

Pathway enrichment analysis was performed using Gene Ontology (GO, RRID:SCR_002811) and KEGG (RRID:SCR_027172)^17^ databases, focusing specifically on sub-cluster 2. Analyses were performed separately for up-regulated and down-regulated DEGs, selecting genes with adjusted p-value < 0.05 and |Log_2_-FoldChange| > 0.25. Pathways were considered significantly enriched when q-value < 0.01, and if composed at least by 3 genes. Results were visualized in R using ggplot2 (v4.0.0, RRID:SCR_014601) as dot plots in which the dot position along the x-axis and the color intensity reflect the -Log_10_(adjusted p-value) (from blue for less significant to red for more significant), while the dot size indicates the number of DEGs in each pathway.

### Cytotoxicity and inflammatory gene set analysis

To evaluate the functional phenotype of both CD56^bright^ and CD56^dim^ NK cells, we computed two module scores relying on publicly available gene signatures^18^, which consider multiple genes simultaneously to provide a comprehensive overview of the functional state of the cells of interest. Specifically, we analyzed two gene sets: a cytotoxicity signature (*GZMA*, *GZMB*, *GZMH*, *GZMM*, *GZMK*, *GNLY*, *PRF1*, *CTSW*) and an inflammatory signature (*CCL2*, *CCL3*, *CCL4*, *CCL5*, *CXCL9*, *CXCL10*, *IL1B*, *IL6*, *IL7*, *IL15*, *IL18*). Gene set scores were calculated with the *scanpy.tl.score_genes()* function, as previously described^19^.

### Statistical analysis

Statistical analyses were performed using GraphPad PRISM software (v10.1.1, RRID:SCR_002798). The significance of data between DEX-treated and untreated patients was evaluated using the Mann-Whitney test. P-values were considered statistically significant when p < 0.05 and were represented by the following symbols: *p < 0.05, **p < 0.01, ***p < 0.001, ****p < 0.0001.

## Results

### Perioperative corticosteroid treatment was associated with variations in the intratumoral distribution of NK cell subsets in gliomas

To characterize the transcriptional profile of glioma-infiltrating NK cells, we analyzed a publicly available single-cell RNA sequencing dataset obtained from FACS-sorted viable CD45⁺ cells isolated from five IDH-WT diffuse glioma lesions and two non-tumor brain tissue specimens (**Material and Methods**). Among the five IDH-WT glioblastoma patients, two received dexamethasone (DEX) before surgery, whereas the remaining three were naïve to corticosteroid treatment.

Feature-barcode matrices from each sample were converted into Python AnnData objects and integrated with the Harmony algorithm, resulting in a dataset of 27462 cells, subdivided into 20 clusters (Supplementary Figure 1A). Among the CD3^neg^ cells, NK cells were identified in cluster 12 based on the expression of well-known NK cell markers such as *KLRB1*, *KLRF1*, *CD247*, *CD7* and *FCGR3A* (Supplementary Figures 1A-B)*.* This annotation was further validated by applying a gene signature score confirming the identity of NK cells according to a combination of established marker expression profiles^20^ (Supplementary Figure 1C).

**Fig. 1:**
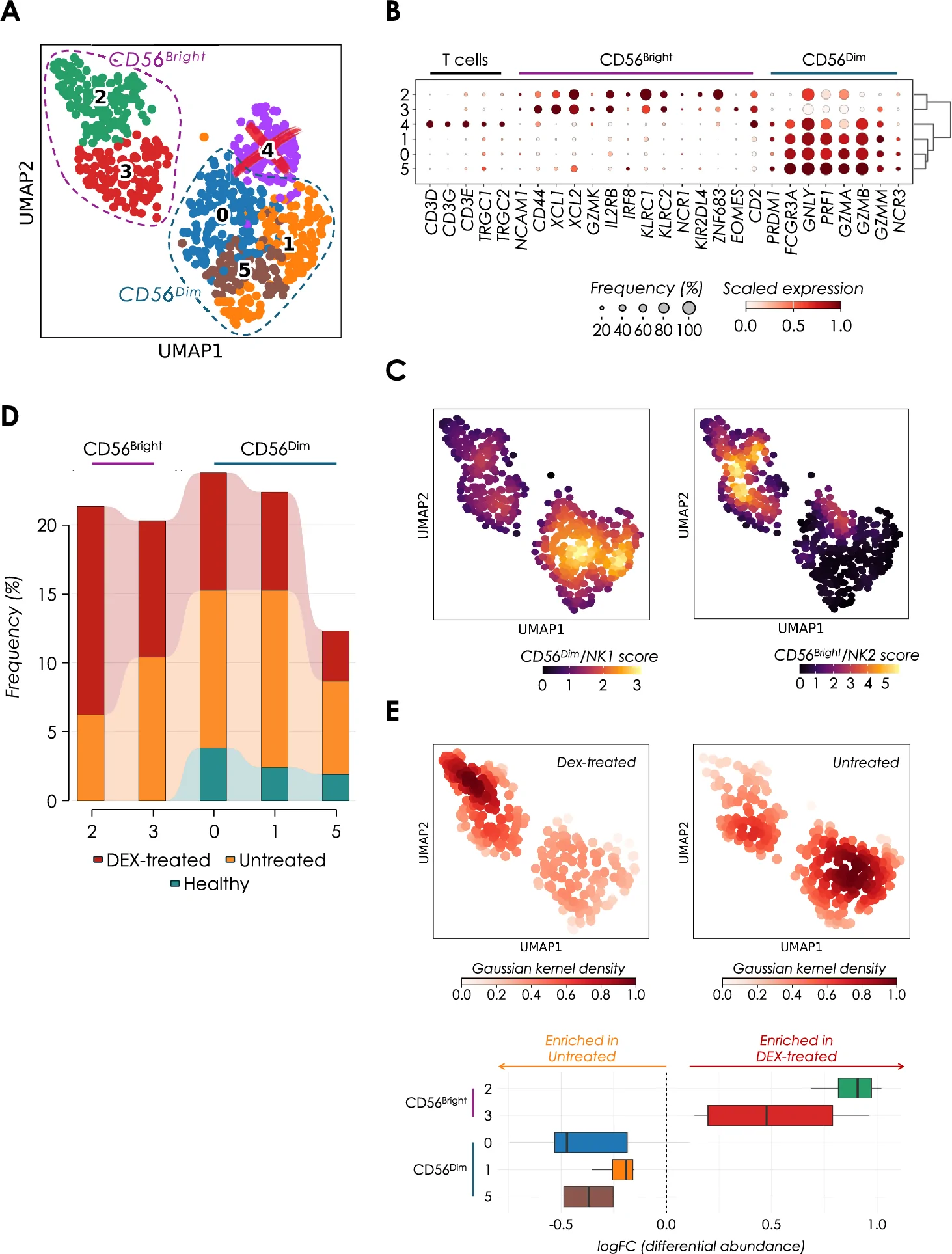
Perioperative corticosteroid treatment is associated with variations in intratumoral distribution of NK cell subsets. (**A**) UMAP clustering projection of the integrated glioma-associated NK cells from all subjects. (**B**) Dot plot showing the expression of canonical gene markers for the annotation of NK cell subsets. (**C**) Kernel density of NK1 and NK2 gene set scores embedded on UMAP projection. (**D**) Bar plot showing the proportion of cells in each sub-cluster deriving from healthy tissue, DEX-treated tumors or untreated tumors. (**E**) Top panel: density plots showing the distribution of NK cells from DEX-treated and untreated patients on UMAP projections. Bottom panel: boxplots showing the distribution of differential abundance values calculated by Milopy across neighborhoods assigned to each NK cell sub-cluster. Positive and negative log fold change (logFC) values indicate neighborhoods relatively enriched and depleted in DEX-treated patients compared with untreated patients, respectively. The dashed vertical line marks LogFC = 0, corresponding to no change between conditions. Statistical significance was assessed at the neighborhood level using Milopy spatial FDR correction.

To refine the characterization of NK cells and better dissect their cellular heterogeneity, we performed a cell reclustering of cluster 12, and we identified six distinct sub-clusters (Fig. 1A). Sub-cluster 4 was identified as T cells, likely γδ T cells, based on the high expression of *CD3D*, *CD3E*, *CD3G* and of the T cell receptor γ chain genes *TRGC1* and *TRGC2* and was therefore excluded from further analyses (Fig. 1B). This contamination is consistent with previous reports describing the strong transcriptomic similarity between NK cells and γδ T cells in scRNA-seq datasets^21,22^. Sub-clusters 2 and 3 were annotated as CD56^bright^ NK cells expressing high levels of *NCAM1*, *XCL1*, *IL2RB*, *KLRC1* genes together with other genes described to be associated with CD56^bright^ NK cells^20^ (Fig. 1B and Supplementary Figure 2). Similarly, sub-clusters 0, 1, and 5 were annotated as CD56^dim^ NK cells based on the high expression of *FCGR3A*, *PRF1* and granzymes genes (Fig. 1B and Supplementary Figure 2). To further support our supervised annotation, we applied a recently published gene signature scoring approach that discriminates between NK1 (CD56^dim^) and NK2 (CD56^bright^) subsets^20^. This analysis confirmed the supervised identity of CD56^dim^ and CD56^bright^ NK cells in our dataset (Fig. 1C).

The analysis of relative NK cell distribution revealed that, in healthy brain tissue samples, NK cells were markedly underrepresented compared with glioma lesions, as expected^23,24^. They were restricted to the CD56^dim^ NK cell clusters, whereas CD56^bright^ NK cell subsets were completely absent (Fig. 1D). In glioma patients, we observed a different distribution of NK cell subsets according to perioperative corticosteroid treatment. In particular, DEX-treated patients showed a significant shift toward CD56^bright^ NK cells compared with untreated patients, evidenced by a marked enrichment of sub-cluster 2 (Figs. 1D-E).

### CD56^***bright***^ sub-cluster 2 showed the strongest transcriptional changes associated with corticosteroid exposure

**Fig. 2:**
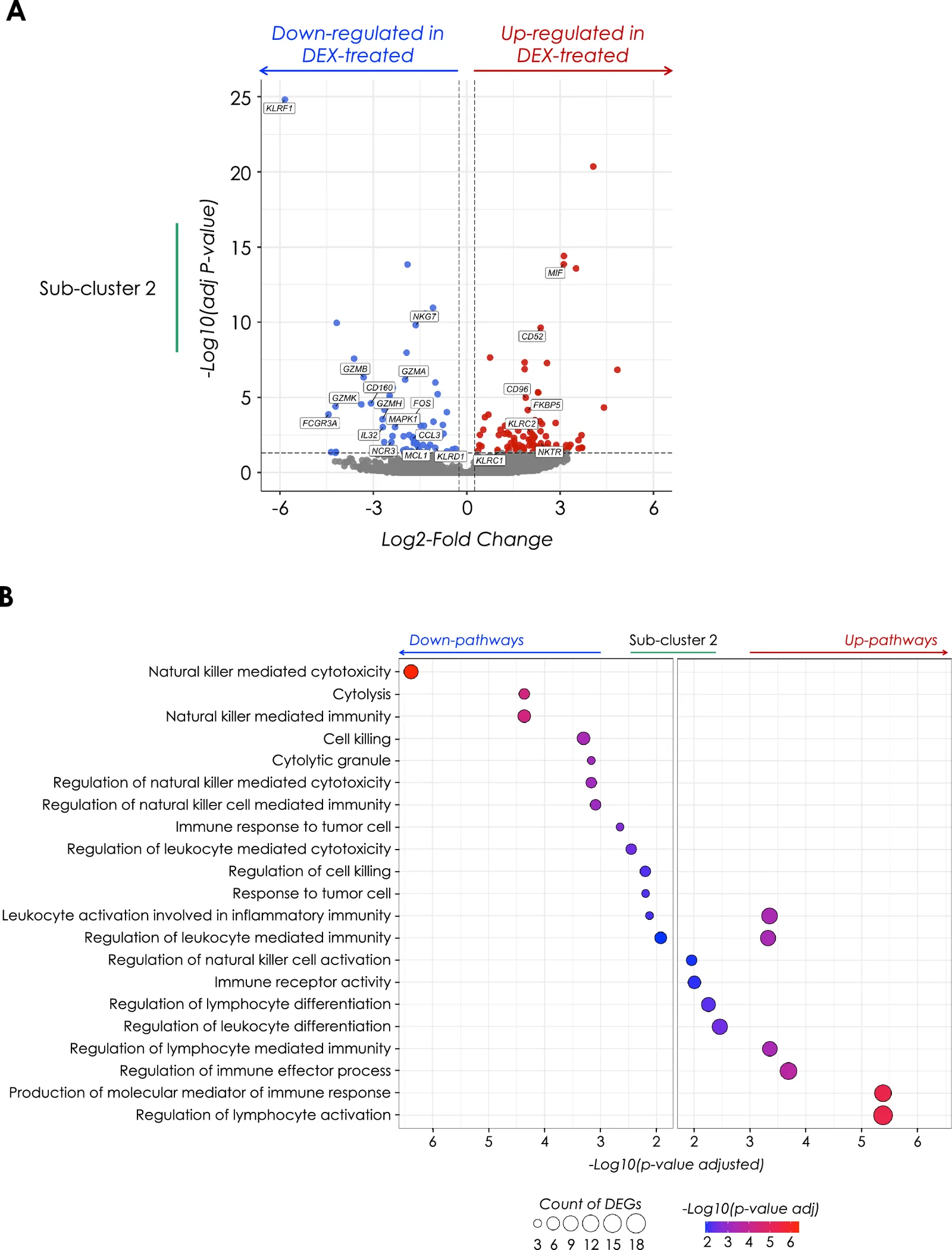
CD56^bright^ NK cells exhibit the strongest transcriptional changes associated with corticosteroid exposure. (**A**) Volcano plot showing DEGs between DEX-treated and untreated patients in sub-cluster 2. DEGs were defined as genes with |Log_2_ FC| ≥ 0.25 and adjusted p-value < 0.05. (**B**) Dot plot showing the negatively and positively enriched GO and KEGG^17^ pathways in sub-cluster 2 of DEX-treated versus untreated patients. The x-axis and the color of each dot represent the -Log_10_(adjusted p-value), while their size indicates the number of DEGs in each pathway.

To investigate the association between corticosteroid treatment and glioma-infiltrating NK cell transcriptional programs, we performed a differentially expressed gene (DEG) analysis comparing cells from DEX-treated and untreated patients within each NK cell sub-cluster. Sub-cluster 2 showed the highest number of significant DEGs (n = 180), whereas sub-clusters 0, 1, 3, and 5 showed only few or no DEGs (3, 0, 12, and 0 respectively) (Supplementary Table 1). Therefore, downstream analyses were focused on sub-cluster 2. In this sub-cluster, NK cells from DEX-treated patients showed a significant down-regulation of key mediators of NK cell activity, including the chemokine *CCL3*, the cytotoxic effector *NKG7*, multiple granzymes (*GZMA*, *GZMK*), and the activating receptor *NCR3* (Fig. 2A). Conversely, *FKBP5* was among the most up-regulated genes in DEX-treated patients, consistent with its role as a glucocorticoid-inducible regulator of steroid receptor signaling (Fig. 2A)^25,26^. Other up-regulated DEGs included *CD96*, *MIF*, *CD52*, and *KLRC2*, encoding for an immune checkpoint receptor, an immunoregulatory cytokine, a lymphocyte surface glycoprotein, and the activating receptor *NKG2C,* respectively. To deeply investigate the biological functions associated with sub-cluster 2, all DEGs were subjected to enrichment functional analysis (gene ontology, Log_2_FC-weighted), which highlighted a significant down-regulation of immune-related pathways in DEX-treated compared with untreated patients. Pathways associated with NK cell effector functions showed the strongest enrichment among down-regulated pathways. The most affected pathways included NK cell-mediated cytotoxicity, cytolysis, cell killing, and cytolytic granule activity. Consistently, additional immune-related categories such as immune response to tumor cells and regulation of NK cell-mediated cytotoxicity were also enriched among down-regulated pathways (Fig. 2B).

### Corticosteroid-associated transcriptional changes were detectable in all sub-clusters of glioma-infiltrating NK cells

**Fig. 3:**
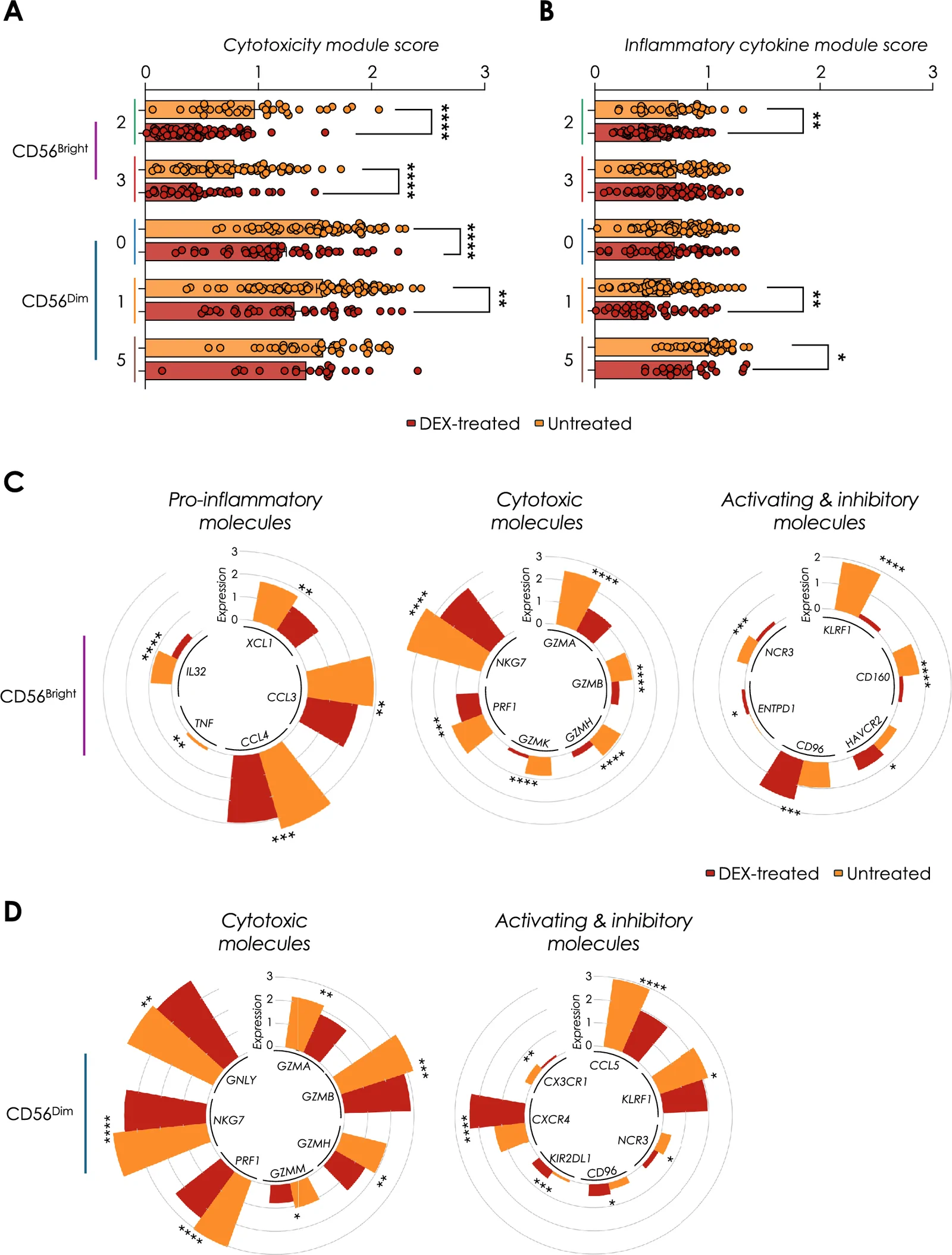
Corticosteroid treatment is associated with reduced cytotoxic and inflammatory transcriptional programs in glioblastoma-infiltrating NK cells. (**A**) Bar plot showing the cytotoxicity score values across sub-clusters, in DEX-treated and untreated patients. (**B**) Bar plot showing the inflammation score values across sub-clusters, in DEX-treated and untreated patients. (**C**) Circular bar plots showing the mean normalized expression level of selected genes in CD56^bright^ NK cells from DEX-treated and from untreated patients. (**D**) Circular bar plots showing the mean normalized expression level of selected genes in CD56^dim^ NK cells from DEX-treated and from untreated patients. Statistical significance was computed with Mann-Whitney test (*p < 0.05, **p < 0.01, ***p < 0.001, ****p < 0.0001).

In order to support and strengthen the relevance of the results obtained by DEG analysis, we further analyzed single-cell RNA data by applying previously described gene signatures to compute cytotoxicity and inflammation scores in each NK cell subset (**Material and Methods**). By capturing the coordinated expression of multiple effector molecules rather than relying on individual genes, this approach has the advantage to provide a more comprehensive, statistically powerful, and interpretable view of complex traits by integrating information from many genes. This approach allowed us to explore functional alterations across all subsets. Scores were then compared between DEX-treated and untreated patients to assess whether corticosteroid exposure differentially affected the cytotoxic and inflammatory programs of intratumoral NK cells. Indeed, we observed that within each NK cell cluster, cells from DEX-treated patients displayed significantly lower functional scores compared with those from untreated patients. In particular, sub-clusters belonging to both CD56^bright^ (sub-clusters 2 and 3) and CD56^dim^ NK cell subsets (sub-clusters 0 and 1) from DEX-treated patients showed a marked reduction in cytotoxicity scores (Fig. 3A). Similarly, sub-clusters belonging to both NK cell subsets (namely, sub-clusters 2, 1, and 5) also showed significantly reduced inflammation scores in DEX-treated patients compared with untreated patients (Fig. 3B).

To further assess whether the transcriptional changes associated with dexamethasone exposure were detectable across all sub-clusters of glioblastoma-infiltrating NK cells, we analyzed individual genes underpinning the cytotoxicity and inflammation scores and observed, indeed, that the expression of relevant cytotoxic molecules and inflammatory cytokines was significantly reduced in DEX-treated patients across all sub-clusters (Supplementary Figures 3A-B).

The same results were even more evident when the same individual genes were analyzed grouping sub-clusters 2 and 3 as CD56^bright^ NK cells, and sub-clusters 0, 1 and 5 as CD56^dim^ NK cells, thus highlighting consistent association between dexamethasone exposure and transcriptional changes of the two main NK cell subsets (Figs. 3C-D). Indeed, CD56^bright^ NK cells from DEX-treated patients showed lower expression of genes encoding for the inflammatory cytokines *XCL1*, *CCL3*, *CCL4*, *TNF*, and *IL32*, the cytotoxic effector molecules *GZMA*, *GZMB*, *GZMH*, *GZMK*, *PRF1*, and *NKG7*, and the activating receptors *KLRF1*, *NCR3*, and *CD160* compared with CD56^bright^ NK cells from untreated patients (Figure 3C). This reduction was more pronounced in sub-cluster 2 (Supplementary Figure 3A). A similar pattern was observed in CD56^dim^ NK cells. Indeed, CD56^dim^ NK cells from DEX-treated patients exhibited significantly lower expression of genes encoding for cytotoxic effector molecules *GZMA*, *GZMB*, *GZMH*, *GZMM*, *PRF1*, *GNLY*, and *NKG7* and activating receptors *KLRF1*, *NCR3*, and *CX3CR1*, together with higher expression of genes encoding for inhibitory molecules such as *CD96*, *KIR2DL1*, and *CXCR4*, compared with CD56^dim^ NK cells from untreated patients (Figure 3D). This reduction was more pronounced in sub-cluster 0 (Supplementary Figure 3B).

## Discussion

In this study, we provide single-cell transcriptomic evidence suggesting that perioperative corticosteroid treatment profoundly affects glioblastoma-infiltrating NK cells. By analyzing scRNA-seq data from IDH-WT glioblastoma patients treated or not with dexamethasone, we observed a perturbation in NK cell subset composition, a broad suppression of cytotoxic and inflammatory programs, and the upregulation of inhibitory pathways, with CD56^bright^ NK cells exhibiting the most pronounced dysfunction.

Our results suggest that corticosteroid exposure is associated with a reshaped intratumoral NK cell landscape, leading to a significant enrichment of CD56^bright^ subsets, typically associated with cytokine production and immunoregulatory functions rather than direct cytotoxicity^27^. Therefore, the relative enrichment of CD56^bright^ NK cells in DEX-treated patients may suggest that perioperative corticosteroid treatment may affect the intratumoral composition fostering the enrichment of subsets with limited anti-tumor potential. Consistently, CD56^bright^ NK cells were also the most affected subset by corticosteroids. Notably, one cluster of CD56^bright^ NK cells showed the highest responsiveness to DEX treatment, as assessed by the higher number of differentially expressed genes. Accordingly, this cluster also up-regulated *FKBP5*, a glucocorticoid-inducible gene involved in the negative feedback regulation of steroid receptor signaling^25,26^, further supporting a strong association between corticosteroid treatment and NK cell transcriptional changes observed in DEX-treated patients.

Although CD56^bright^ NK cells represented the most affected subset, corticosteroid treatment was associated with a broader impairment of NK effector programs. Indeed, multiple gene scores demonstrated a significant reduction of both cytotoxicity and inflammation features across CD56^bright^ and CD56^dim^ NK cell clusters in DEX-treated patients. This effect was supported by a lower expression of genes encoding for key cytotoxic molecules, including *GZMs*, *PRF1* and *GNLY*, as well as cytokines such as *XCL1*, *CCL3*, *CCL4* and *TNF*. Importantly, gene expression of activating receptors critical for NK cell–mediated target recognition, such as *KLRF1*, *NCR3* and *CD160*^28,29^, was also down-regulated. Notably, tumor-infiltrating NK cells from DEX-treated patients also showed increased expression of genes encoding for inhibitory immune checkpoints, including *HAVCR2*, *CD96*, *ENTPD1*, *KIR2DL1* and *CXCR4*. Although CXCR4 is a chemokine receptor known for its crucial role in cell migration, it has recently been reported also to serve as a novel immune checkpoint receptor on NK cells, capable of restraining NK cell activity and cytotoxic potential^30^. Therefore, the results of our study are consistent with the possibility that corticosteroid exposure simultaneously weakens cytotoxicity, dampens pro-inflammatory potential, and enhances inhibitory signaling, thereby promoting a state of profound NK cell dysfunction.

These results align with and extend previous evidence on the detrimental effects of perioperative corticosteroids on the glioblastoma immune microenvironment. In a previous study, we demonstrated that dexamethasone-treated glioma patients undergo a marked reduction in circulating and tumor-infiltrating dendritic cells (DCs). These cells also showed a transcriptional profile indicative of severe functional impairment, including reduced antigen presentation and costimulatory capacity^31^. Combined with the results presented in this study on NK cells, the emerging scenario may suggest that perioperative corticosteroids may simultaneously weaken both the innate cytotoxic compartment and the professional antigen-presenting arm of anti-tumor immunity, thereby undermining both direct effector functions and the activation of adaptive immune responses.

This study has some limitations, including the small number of patients analyzed, the observational nature of the dataset, the lack of direct functional validation of NK cell activity and its target limited to IDH-WT glioblastoma only. Nevertheless, it provides novel insights into the mechanisms by which corticosteroid treatment may compromise innate immune surveillance. Indeed, NK cells are well recognized key effectors of anti-tumor immunity, directly killing tumor cells and shaping adaptive responses through crosstalk with DCs and T cells^32,33^. By impairing NK cytotoxicity and cytokine-related transcriptional programs, corticosteroids may therefore not only blunt direct tumor killing but also hinder the broader NK cells-mediated orchestration of anti-tumor immunity.

Moreover, whereas CD56^dim^ NK cells are typically considered the main cytotoxic NK cell subset, CD56^bright^ NK cells play critical roles in producing chemokines such as *XCL1*, which are essential for DCs recruitment into tumors^34,35^. It may be therefore hypothesized that the transcriptional changes observed CD56^bright^ NK cells from DEX-treated patients may affect the cellular crosstalk needed to initiate and activate effective adaptive immune responses. These observations may suggest that corticosteroid therapy may contribute to glioblastoma immune evasion not only by acting directly on T cells, as previously demonstrated^36,37^, but also by indirectly weakening the innate-adaptive immune interface via NK cell impairment.

Corticosteroids have been reported to reduce the overall survival and progression-free survival in patients with IDH-WT and IDH-mutated glioma^38^. We suggest that, if confirmed on largest cohorts of patients, the results of this study may provide mechanistic insights into the detrimental effects of corticosteroids in these patients. Accordingly, recent studies have proposed alternative therapeutic strategies for the management of symptomatic peritumoral cerebral edema^39-41^, highlighting the need for safer approaches that control edema while preserving anti-tumor immunity.

## Supplementary Information

Supplementary Information 1.

Supplementary Information 2.

Supplementary Information 3.

Supplementary Information 4.

## Data Availability

scRNA data were downloaded from the Zenodo Repository (RRID:SCR\_004129) where raw data are available upon request. All the codes used for data processing and analysis are available in a public GitHub repository (https://github.com/pmarzano97/GBM.git).
