# Supplementary material for: Single-cell analysis reveals corticosteroid-associated impairment of tumor-infiltrating NK cells in glioblastoma patients
Source: Sci Rep. 2026 May 21;16:26614. doi: 10.1038/s41598-026-52528-1 (PMC13503855; doi:10.1038/s41598-026-52528-1)
Supplement: Supplementary file 1 [file 41598_2026_52528_MOESM1_ESM.pdf]

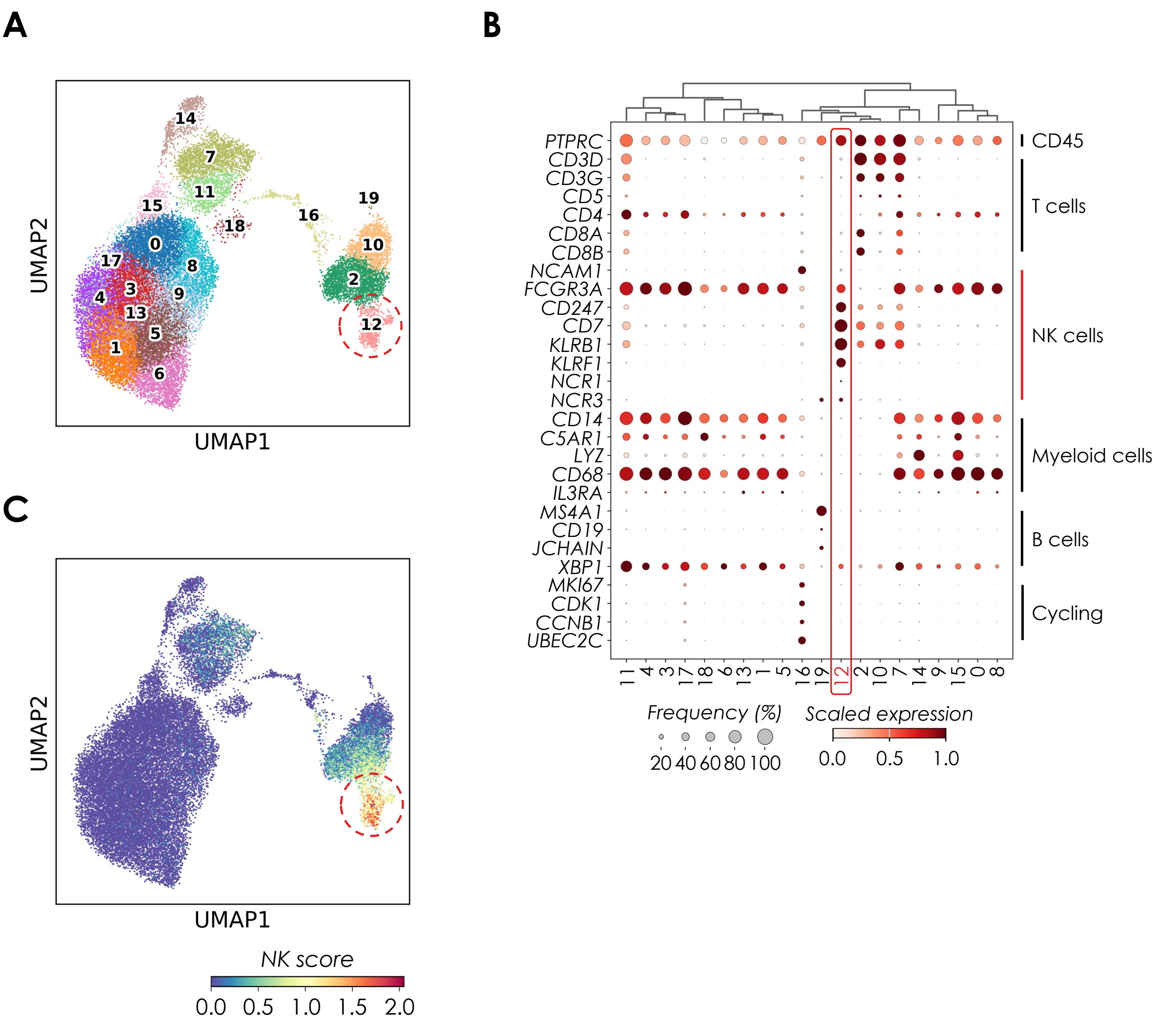

Supplementary Figure 1 | Annotation of IDH-WT glioma-associated CD45⁺ cells.
(**A**) UMAP clustering projection of the integrated glioma-associated CD45⁺ cells from all subjects. Red circle highlights the NK cells cluster. (**B**) Dot plot showing the expression of canonical gene markers for the annotation of immune cells. (**C**) Feature plot showing the NK cell module scores based on 13 genes characteristic of human NK (41).
