# Supplementary material for: Single-cell analysis reveals corticosteroid-associated impairment of tumor-infiltrating NK cells in glioblastoma patients
Source: Sci Rep. 2026 May 21;16:26614. doi: 10.1038/s41598-026-52528-1 (PMC13503855; doi:10.1038/s41598-026-52528-1)
Supplement: Supplementary file 2 [file 41598_2026_52528_MOESM2_ESM.pdf]

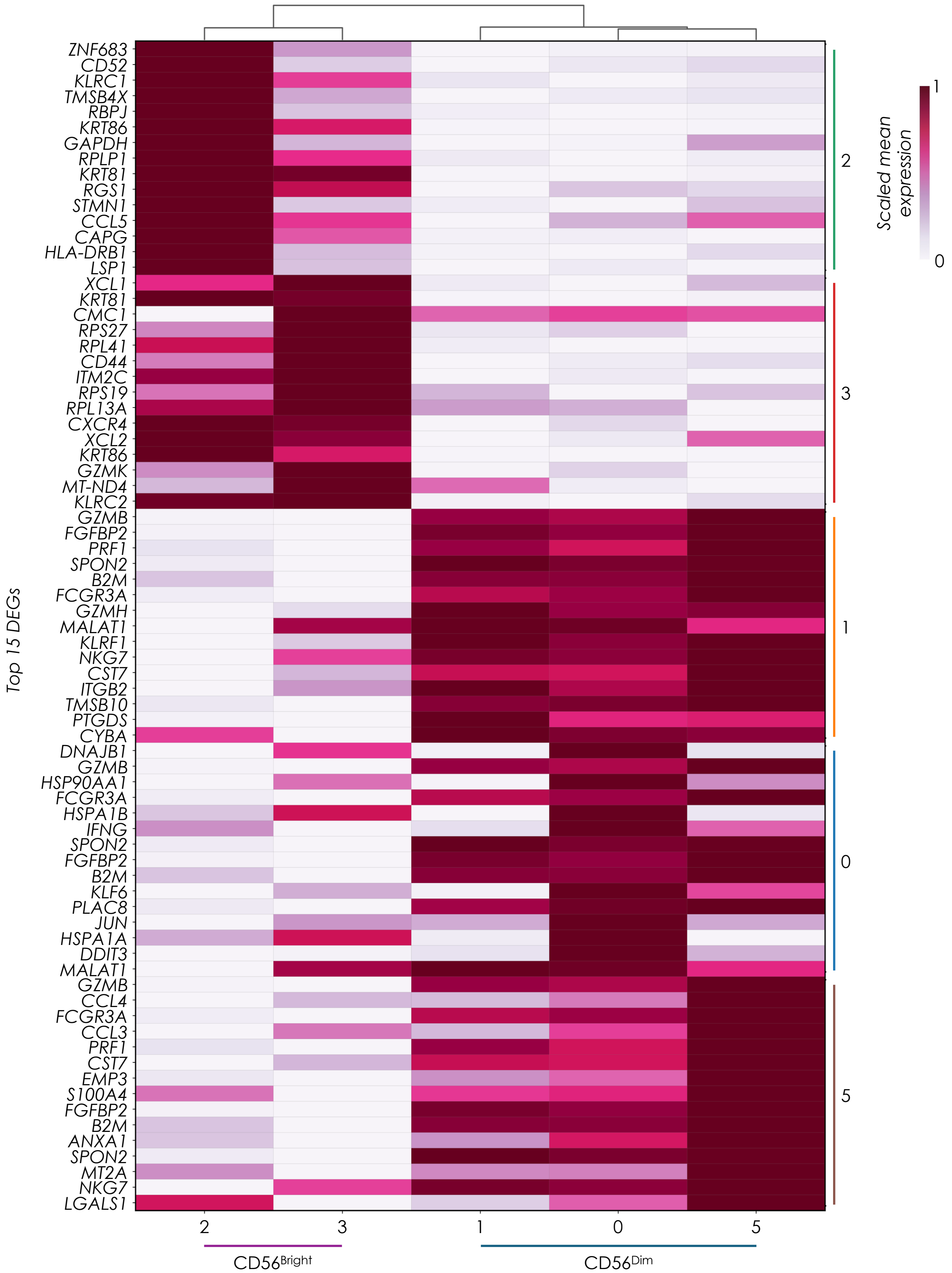

Supplementary Figure 2 | Heatmap showing the top 15 DEGs of each NK cell sub-cluster.
Heatmap showing the scaled gene expression of the top fifteen DEGs identified among NK cell sub-clusters reported on the right.
