# Supplementary material for: Single-cell analysis reveals corticosteroid-associated impairment of tumor-infiltrating NK cells in glioblastoma patients
Source: Sci Rep. 2026 May 21;16:26614. doi: 10.1038/s41598-026-52528-1 (PMC13503855; doi:10.1038/s41598-026-52528-1)
Supplement: Supplementary file 3 [file 41598_2026_52528_MOESM3_ESM.pdf]

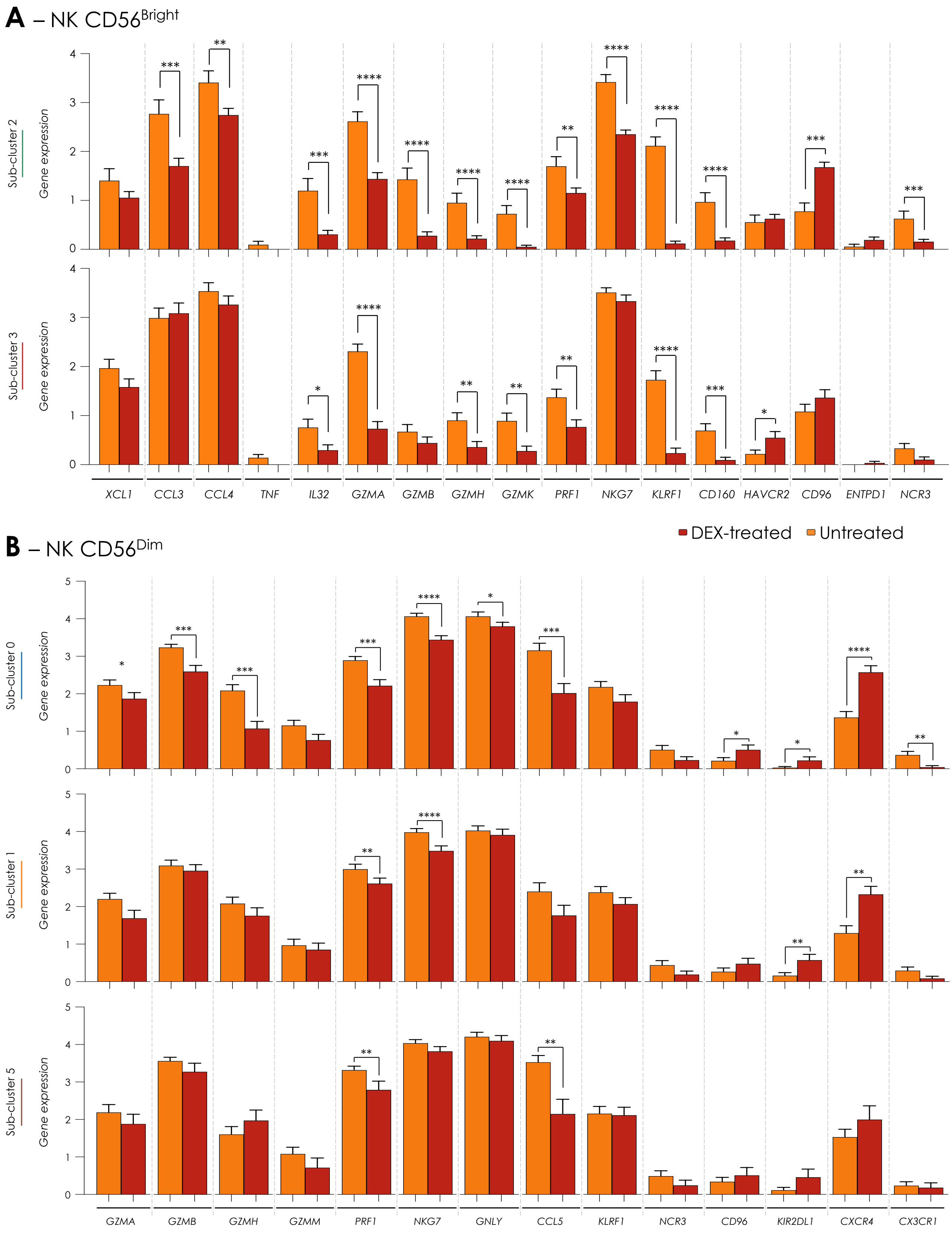

Supplementary Figure 3 | Corticosteroid treatment reduces cytotoxic and inflammatory transcriptional programs in glioma-infiltrating NK sub-clusters.
Bar plots showing the mean normalized expression level of selected genes in CD56bright and CD56dim NK cell sub-clusters from DEX-treated and from untreated patients. Statistical significance was computed with Mann-Whitney test (*$p < 0.05$, **$p < 0.01$, ***$p < 0.001$, ****$p < 0.0001$).
