# Supplementary material for: Single-cell analysis reveals corticosteroid-associated impairment of tumor-infiltrating NK cells in glioblastoma patients
Source: Sci Rep. 2026 May 21;16:26614. doi: 10.1038/s41598-026-52528-1 (PMC13503855; doi:10.1038/s41598-026-52528-1)
Supplement: Supplementary file 4 [file 41598_2026_52528_MOESM4_ESM.pdf]

Reclustering NK cells – DEGs sub-cluster 0 - Dex-treated vs Untreated

Genes	log2foldchanges	pvals	Adjusted pvals
CXCR4	2,169762611	3,87024E-06	0,02707359
RPS27	0,598448038	6,19561E-07	0,006501054
B2M	-0,455357522	2,70769E-09	5,68237E-05

Up Dex-treated
Up Untreated

Reclustering NK cells – DEGs sub-cluster 2 - Dex-treated vs Untreated

Genes	log2foldchanges	pvals	Adjusted pvals
HLA-DRB5	29,5858326	1,76625E-08	1,27815E-05
CAMK4	29,02540588	4,39453E-07	0,00021958
HSPA6	28,9797821	2,13373E-05	0,005394986
PPP3CA	28,76253891	7,08497E-06	0,002360083
STN1	28,67524147	5,43995E-06	0,001902713
DDX1	28,57206726	2,09261E-05	0,00535555
DENND6B	28,48672867	2,16333E-05	0,005404713
SLC25A46	28,46956825	3,94236E-05	0,00919096
PPIL3	28,4192791	7,77184E-05	0,014963287
ZCCHC10	28,36526108	7,6828E-05	0,01492882
FOPNL	28,28669167	6,46421E-05	0,013431471
FAM30A	28,23905945	0,000138639	0,022251393
DNAJB2	28,17690849	6,45549E-05	0,013431471
COPB2	28,16659737	8,9319E-05	0,016159046
PADI4	28,14151382	0,000137	0,022251393
MRPL37	28,13738441	0,000254719	0,034048033
CRACR2B	28,12806129	0,000147204	0,022830676
SUPT6H	28,08245277	0,00014883	0,022830676
PPIP5K2	28,06425095	0,000137428	0,022251393
QPCT	28,05550194	0,000281269	0,03599213
PHF3	28,04741096	0,000273153	0,035733702
ABCB7	27,96550751	0,000403478	0,048110147
GSTM2	27,95454025	0,000427868	0,049884609
FAM214A	27,91610718	0,000407335	0,048295677
SENP7	27,90977859	0,000390927	0,046879973
SRGAP3	4,842256069	1,28128E-10	1,49383E-07
B3GNT7	4,410033703	7,78645E-08	4,80607E-05
RBPJ	4,063755035	4,17748E-25	4,38343E-21
LY9	3,708658695	0,000144068	0,022562775
PHF14	3,689562082	0,000137355	0,022251393
CLNK	3,689254999	1,12345E-05	0,003320677
AAMP	3,597708702	0,000171165	0,025296248
ARHGAP18	3,590643167	3,03255E-05	0,007231935
HLA-DRA	3,51205039	7,60945E-18	2,66153E-14
IFNAR1	3,332256556	7,01887E-05	0,014440983
RGS16	3,279160976	0,000119818	0,02027817
CEP57	3,264591217	0,000135924	0,022251393
SYNE1	3,196576834	8,27858E-05	0,015374713
CCDC28A	3,171557665	0,000296377	0,037244173
ZNF683	3,120104551	5,64904E-19	3,95169E-15
MIF	3,11550808	2,66795E-18	1,39974E-14
TMOD3	3,048994303	0,000245937	0,033298313
FIP1L1	2,878073454	7,54967E-05	0,01492882
MTRNR2L8	2,855627775	1,1626E-06	0,000508297
G3BP1	2,622864246	0,000143649	0,022562775
TRA2A	2,605758667	5,35642E-05	0,011496201
SRSF4	2,589157104	0,000139124	0,022251393
CRIP1	2,580181837	3,99845E-11	5,24447E-08
SNRNP40	2,57448864	0,000155172	0,023488035
CDKN1B	2,56165576	0,00010309	0,018443191
CCND2	2,542746782	1,40593E-05	0,003922868
FABP5	2,517183781	0,000219248	0,030411211
PTP4A1	2,474989414	0,000288276	0,036665158
PCID2	2,415239096	1,37866E-06	0,000590462
YBX1	2,408389807	0,000156628	0,023488035
NKTR	2,382215977	5,27124E-05	0,011496201
CD52	2,376536131	1,22864E-13	2,34402E-10
FNBP1	2,372752428	5,06659E-06	0,001802159
PRKDC	2,351526022	0,000264545	0,034916617
RSL24D1	2,349325657	8,71717E-07	0,000397692
GPR174	2,33106041	0,000274868	0,035733702
ZRANB2	2,304011345	0,000131299	0,022043479
PIK3R1	2,294605732	5,56733E-09	4,86816E-06
PCBP2	2,287445307	5,14394E-09	4,69351E-06
WIPF3	2,274353027	0,000156691	0,023488035
TMEM183A	2,270133018	0,000275844	0,035733702
C12orf75	2,262056112	0,00011626	0,020163862
MCTP2	2,211585522	0,000208423	0,029355442

Up Dex-treated
Up Untreated

CAPG	2,191753387	7,13086E-07	0,000332551
NAP1L4	2,178683281	1,57418E-05	0,004235341
TENT5C	2,16386795	8,25006E-05	0,015374713
PPP1CB	2,162158012	1,90321E-05	0,004992597
PRPF38A	2,12418437	0,000354606	0,043015911
PIN1	2,103621244	4,38421E-05	0,009787989
TBCB	2,103172302	8,68151E-06	0,002760458
TRABD	2,091614008	8,06695E-05	0,015374713
KLRC2	2,042743444	6,10395E-06	0,002099959
CTSA	2,036157608	2,59313E-05	0,006327843
HSPD1	2,005346775	8,75164E-05	0,015970598
PEBP1	1,993263006	2,69126E-05	0,006491806
FKBP5	1,962900519	1,1951E-07	6,96678E-05
CD96	1,892969251	1,35377E-08	1,05223E-05
PABPC1	1,8611058	1,07883E-10	1,33178E-07
PTPRC	1,861010671	3,37107E-11	4,71635E-08
MACF1	1,837077737	0,000220266	0,030411211
SET	1,820430875	0,000181737	0,026485619
PRRC2C	1,81888628	1,48973E-05	0,00406019
SERP1	1,802049994	0,000113862	0,019912492
VAPA	1,685507536	7,67438E-05	0,01492882
NDUFB1	1,671628356	0,000188514	0,027283837
HLA-DPB1	1,656373024	4,54186E-06	0,001643371
PARP8	1,653947115	0,000326724	0,040571745
ICAM3	1,583028913	0,000118447	0,02027817
EIF4G2	1,577878475	0,000206237	0,029243889
SRRM1	1,544315338	8,62738E-05	0,015881945
HLA-DRB1	1,526382089	3,43634E-05	0,008102819
OSTF1	1,518927455	1,93342E-05	0,005009238
ITGB1	1,419836521	5,08365E-05	0,011230059
HOPX	1,36047709	9,34038E-06	0,002925632
ELF1	1,360403895	0,000191036	0,027459512
BRK1	1,314361572	0,000420847	0,049591849
TMIGD2	1,306522012	6,97438E-06	0,002360083
SLC25A5	1,304779768	0,000119146	0,02027817
RPS26	1,232385993	1,86466E-06	0,00076729
RPL4	1,224483013	8,3708E-06	0,002702609
CD74	1,08536911	3,89662E-06	0,001434641
RPLP0	1,034134388	1,06879E-05	0,003204225
KLRC1	1,007048249	0,000341062	0,041856931
RPS17	0,990525723	0,000236022	0,032163362
RPS29	0,748943686	1,40738E-11	2,27194E-08
RPS2	0,695175827	2,66653E-07	0,000143487
RPS27	0,584355652	4,14136E-07	0,000211977
RPLP1	0,533127189	3,87098E-06	0,001434641
RPL39	0,474024445	0,000103702	0,018443191
PTMA	0,424076408	1,01531E-05	0,003133432
RPL21	0,406836808	8,19514E-05	0,015374713
RPL15	0,363980383	0,000279952	0,03599213
MALAT1	-0,368360341	0,000181105	0,026485619
MT-CO2	-0,455147535	0,000215551	0,030157092
H3F3B	-0,633622468	0,000314095	0,039235751
HLA-A	-0,637883961	1,72026E-07	9,75711E-05
MT-CO3	-0,744250238	8,06366E-06	0,002644124
EIF1	-0,767577112	1,62854E-06	0,00068353
HCST	-0,840035319	1,40684E-05	0,003922868
FAU	-0,932679772	7,29369E-09	6,12261E-06
MT-ATP6	-1,009391904	1,03865E-09	1,03796E-06
KLRD1	-1,012453914	0,000149042	0,022830676
MYL12A	-1,070139408	9,34459E-07	0,000417246
HLA-B	-1,084031582	3,6235E-15	1,08633E-11
LY6E	-1,176687002	7,10242E-05	0,014471008
CD247	-1,255158424	0,000139959	0,022251393
MT-CYB	-1,336623192	1,05285E-14	2,76188E-11
MT-ND1	-1,375389218	1,9398E-06	0,0007791
CNBP	-1,406420708	7,20032E-05	0,01452942
ZFP36	-1,461359501	2,80972E-08	1,96549E-05
IFITM2	-1,479314327	1,96761E-06	0,0007791
ITGB2	-1,50381422	0,000330974	0,040857729
MYDGF	-1,518467903	0,000264088	0,034916617
MCL1	-1,560855985	0,000106937	0,018858618

YPEL5	-1,594396353	7,49035E-05	0,01492882
LIMD2	-1,605426073	5,45749E-05	0,011568779
NKG7	-1,637289166	7,43991E-14	1,56134E-10
CYCS	-1,680954814	3,98541E-05	0,00919096
GPSM3	-1,699728847	5,36847E-05	0,011496201
BCAP31	-1,718642592	1,71218E-05	0,004548318
FOS	-1,721210957	4,70905E-07	0,000229823
CCL3	-1,733626366	2,23818E-05	0,00552594
APMAP	-1,777647257	0,000293748	0,037136157
UCP2	-1,847408533	1,06637E-05	0,003204225
MT-ND2	-1,902641892	3,46689E-18	1,45512E-14
CIRBP	-1,930595875	6,11581E-12	1,06955E-08
LBH	-1,934899092	0,000202932	0,028970985
GZMA	-1,976460218	6,39595E-10	6,71127E-07
PHB	-2,027383804	1,42065E-05	0,003922868
GIMAP7	-2,037102699	0,000248082	0,033373358
ARPC5L	-2,123684645	3,60324E-07	0,000189044
MAPK1	-2,30278945	2,5212E-06	0,000961999
CST7	-2,376231194	2,44876E-09	2,33589E-06
IER2	-2,38389039	1,31733E-05	0,003787059
NCR3	-2,420536757	4,27023E-05	0,009636036
SPON2	-2,441443205	0,000223392	0,030641269
ITM2C	-2,475123405	9,46944E-09	7,6433E-06
AC004687.1	-2,477992296	1,58584E-08	1,18859E-05
TUBA4A	-2,494932175	0,000160175	0,023839944
ARL4C	-2,646312237	1,0974E-07	6,58002E-05
PTGER2	-2,6507411	4,17695E-05	0,009527988
IL32	-2,694561481	2,49063E-06	0,000961999
GZMH	-2,705772161	6,02735E-07	0,000287477
CD160	-3,075324774	3,69161E-08	2,4991E-05
GIMAP4	-3,308957815	1,26959E-05	0,003700492
GZMB	-3,311588526	4,25043E-10	4,69472E-07
CMC1	-3,38362956	4,52824E-08	2,96968E-05
TRBC1	-3,618913651	1,7855E-11	2,67646E-08
AC020916.1	-4,178050995	4,80964E-14	1,1215E-10
SMPD3	-4,201451778	0,000350646	0,042782837
GZMK	-4,213103771	6,40893E-08	4,07569E-05
SLC37A4	-4,217967987	0,000422993	0,049591849
AL627171.1	-4,357343197	0,000368722	0,04447129
FCGR3A	-4,439709663	2,48847E-07	0,000137429
KLRF1	-5,83924818	7,3117E-30	1,53443E-25

Reclustering NK cells – DEGs sub-cluster 3 - Dex-treated vs Untreated

Genes	log2foldchanges	pvals	Adjusted pvals
MTRNR2L8	4,482574463	2,35022E-06	0,007045947
IFITM1	2,371924639	1,49798E-05	0,028578791
RPS26	1,642851949	7,07189E-06	0,016490075
RPS29	0,664169073	2,79589E-05	0,048895429
RPS27	0,549197733	8,62545E-08	0,000603379
HLA-B	-0,913997591	6,62322E-06	0,016490075
ACTB	-0,985577643	1,03856E-05	0,021795313
MT-ND2	-1,472106576	5,56653E-07	0,002759614
FOS	-2,127527714	6,57489E-07	0,002759614
KLRC1	-2,383455992	1,56613E-06	0,005477803
GZMA	-3,072017431	4,38542E-11	9,20325E-07
KLRF1	-4,120478153	9,79061E-10	1,02733E-05

Up Dex-treated
Up Untreated
